# Biologically Inspired Information Processing and Synchronization in Ensembles of Non-Identical Threshold-Potential Nanostructures

**DOI:** 10.1371/journal.pone.0053821

**Published:** 2013-01-22

**Authors:** Javier Cervera, José A. Manzanares, Salvador Mafé

**Affiliations:** Facultat de Física, Universitat de València, Burjassot, València, Spain; National Research & Technology Council, Argentina

## Abstract

Nanotechnology produces basic structures that show a significant variability in their individual physical properties. This experimental fact may constitute a serious limitation for most applications requiring nominally identical building blocks. On the other hand, biological diversity is found in most natural systems. We show that reliable information processing can be achieved with heterogeneous groups of non-identical nanostructures by using some conceptual schemes characteristic of biological networks (diversity, frequency-based signal processing, rate and rank order coding, and synchronization). To this end, we simulate the integrated response of an ensemble of single-electron transistors (SET) whose individual threshold potentials show a high variability. A particular experimental realization of a SET is a metal nanoparticle-based transistor that mimics biological spiking synapses and can be modeled as an integrate-and-fire oscillator. The different shape and size distributions of nanoparticles inherent to the nanoscale fabrication procedures result in a significant variability in the threshold potentials of the SET. The statistical distributions of the nanoparticle physical parameters are characterized by experimental average and distribution width values. We consider simple but general information processing schemes to draw conclusions that should be of relevance for other threshold-based nanostructures. Monte Carlo simulations show that ensembles of non-identical SET may show some advantages over ensembles of identical nanostructures concerning the processing of weak signals. The results obtained are also relevant for understanding the role of diversity in biophysical networks.

## Introduction

The route to functional nanoelectronics is mined with weak signals, thermal noise and significant diversity between nominally identical units. This hardware variability may be undesirable for most applications. In particular, the threshold voltage mismatching of individual electronic transistors constitutes a serious problem in voltage-driven applications [1] and nanoscale threshold potential transistors are bound to show a significant heterogeneity in their individual characteristics because of the inherent fabrication uncertainties [2]. The inability to produce significant amounts of identical nanostructures is a major concern for recent developments of silicon-based CMOS circuits [3]. A high variability deteriorates manufacturing yields and individual device reliability [4], being considered a source of potential errors. On the contrary, biological diversity is present in most natural systems [5]–[9]. It is thus of great interest to incorporate biomimetic concepts in alternative information processing schemes based on the integration of heterogeneous nanoscale devices.

*Is it feasible to implement potentially useful information processing schemes with individually different nanostructures?* We show here by means of numerical simulations that this could still be possible for the case of nanostructures with different threshold responses that mimic some of the characteristics of heterogeneous neural populations. It has recently been noted that natural variability can make noisy biological networks to function more efficiently by exploiting the integration of non-identical units in summing arrays [5]–[7]. The interplay between noise and neural heterogeneity produces robust population responses [5], [6]. Although neuronal heterogeneity was originally considered a consequence of biological limitations, the fact is that it provides a wide range of spiking strategies for coding [7], [10], [11]. Intrinsic neuronal diversity can thus be regarded as a potentially useful strategy and not simply as the result of natural imprecision [7], [10].

Frequency-based signal processing is characteristic of the neural populations in the brain and concerns the transduction of external information into patterns of neural activity, discernible rhythms, and synchronization processes [7], [10]–[14]. The commonly used *rate* coding employs the average rates of spikes trains to codify the external stimuli while the *rank order* coding is based on assigning an order to the particular neurons that respond to these stimuli. The rank order coding should be implemented rather easily in biological structures being fast enough to account for the reduced time response of neurons to visual stimuli [13]. Also, the ability of the brain to modulate the properties of different neuron populations and adjust their spiking rhythms under an external stimulus (*synchronization*) can be related to natural communication [14].

We explore here different information processing schemes with heterogeneous groups of non-identical nanostructures that make use of the above biological concepts (diversity, rate and rank order coding, synchronization). To this end, we simulate the integrated response of ensembles of single-electron transistors (SET) whose threshold potentials suffer from a high individual variability [15], [16]. A particular experimental realization of a SET is the nanoparticle-based transistor [17]–[20] that mimics some of the spiking properties of biological synapses [15], [17], [21]. In these systems, the different shape and size distributions of metallic nanoparticles protected with organic ligands result in a significant variability in their threshold potentials [15], [22]. Monte Carlo simulations with these threshold nanostructures have recently shown that moderate redundancy not only decreases the adverse effects of variability and but also enhances the processing of weak, sub-threshold signals by taking advantage of the different individual characteristics [15], [23].

This work builds and extends upon recent studies by us on signal processing schemes using heterogeneous [16] and identical [24] nanostructures. We develop further the frequency-based scheme proposed in a previous letter [16] by comparing the rate and rank order coding schemes for image processing. The emerging collective properties of ensembles with heterogeneous SET are shown to be useful for image reconstruction. Then, we analyze with detail how the nanostructure variability can improve the processing of time dependent analog signals. Finally, we extend our preliminary work on the synchronization of SET ensembles connected to a common coupling element [24] and discuss the effects of heterogeneity. Taken together, the three case studies permit to understand the effects of nanostructure variability in a broad range of information processing schemes that are also relevant for neural networks.

In all cases, we use a mixed continuum-Monte Carlo approach where the nanostructure ensembles are not modeled as replicates of the same unit with constant physical properties but as statistical distributions of physical parameters characterized by average and width distribution values. A significant novelty with respect to previous work [16], [24] is the effort made to connect the effects of diversity in biophysical networks with those arisen from the nanostructure variability, trying to identify the biological concepts that could be useful for implementing signal processing schemes with artificial heterogeneous ensembles.

## Methodology

### Experimental SET

Models describing how neural spike trains convey sensory information can be relatively complex [12], [25]. On the contrary, SET models are relatively simple, being reminiscent of the commonly used integrate-and-fire neuron approach [26]. The simulation procedures described here are based on previous theoretical and experimental studies [15], [17], [23], [27] showing that equivalent electrical circuits can be used to model heterogeneous ensembles of SET and nanowire field-effect transistors. It has been firmly established [15]–[17], [27] that the dynamic range of these circuits widens due to the threshold variability of the individual nanostructures and the non-linear summation process producing the final output.

**Figure 1:**
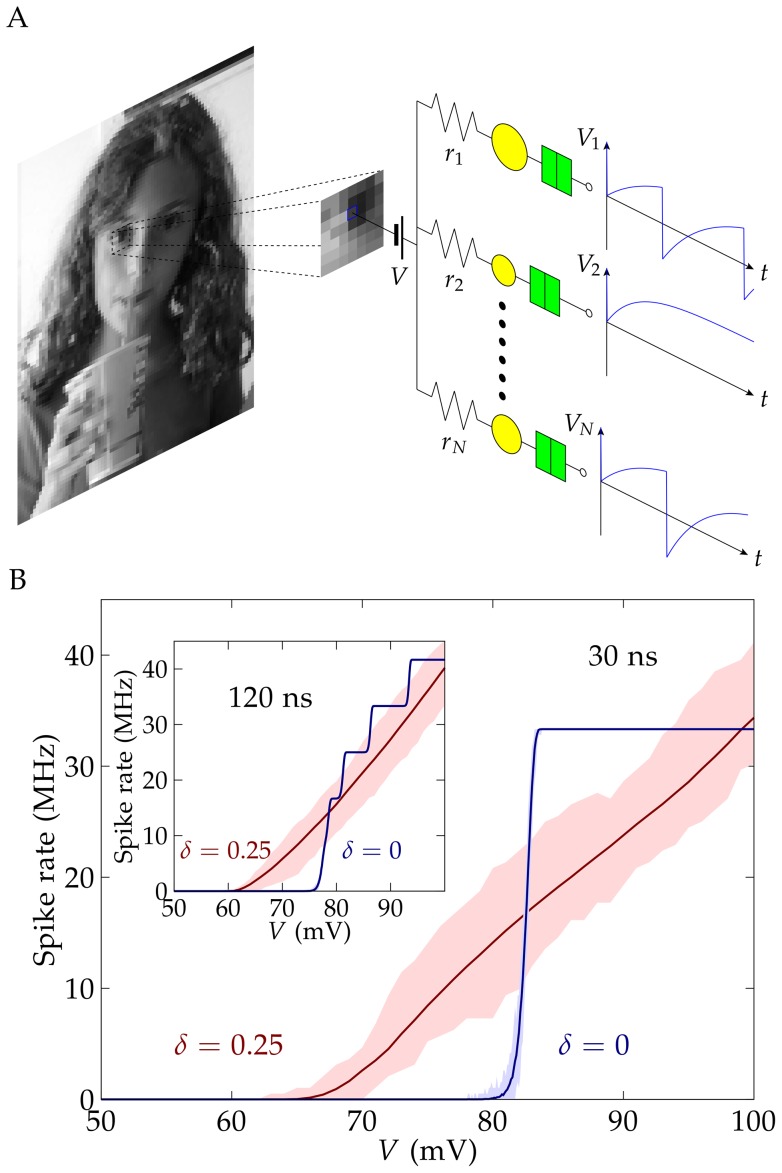
Data processing with an ensemble of R-SETs. The basic information processing unit is an ensemble of *N* R-SET The spherical, ligand-protected metallic nanoparticle (NP) is connected in series to a charging resistance (left) linked to an electrode at potential *V*. The NP consists of a capacitance and a tunnel junction (right) linked to a grounded electrode through a ligand [15]. The potential *V_i_*(*t*) of NP *i* shows an oscillatory behavior at sufficiently high input potentials. The average frequency of the R-SET oscillations (spike rate) can be used to process an image (A). Average spike rate in homogeneous (*δ* = 0) and heterogeneous (*δ* = 0.25) ensembles of *N* = 64 R-SET as a function of the applied voltage. The main plot corresponds to a processing time of 30 ns and the inset to 120 ns. The shadow region shows the maximum and minimum values obtained in the simulations (B). The legal guardian has given written informed consent, as outlined in the PLOS consent form, to publication of the photograph in Figs. 1 and 2.

In particular, parallel arrays of resistance-single electron transistors (R-SET) can be used for frequency-based image processing [16]. The equivalent circuit of the SET consists of a capacitance *C_i_* arranged in parallel to a tunnel junction resistance *R_i_* [23]. The resistance *r_i_* is connected in series to the SET to form the R-SET (Fig. 1A). This system can be realized experimentally by means of ligand-stabilized metallic nanoparticles [2], [18], [19], [28], [29]. The nanoparticle (NP) is functionalized with an organic ligand acting as a tunneling junction and the single electron transfers between the NP and the external electrode are determined by the Coulomb blockade and tunneling effects [2], [18], [19], [28], [29]. These electron transfers lead to measurable electric potential changes of the order of 100 mV for effective NP capacitances of the order of 1 aF [2], [19], [22], [28], [29]. For example, the electrochemically determined capacitance of Au_225_ (diameter 1.8 nm) is 0.6 aF approximately [30] and other particles like Pd_40_ (diameter 1.2 nm) show a capacitance as low as 0.35 aF. The current–voltage curves of a single ligand-stabilized NP obtained by scanning tunneling spectroscopy can be described by SET equivalent circuits that give tunneling resistances and effective capacitances in the range 100 MΩ – 10 GΩ and 0.1 – 10 aF, respectively [2], [18]–[20], [22], [28]–[30]. A final, potentially useful characteristic of ligand-stabilized metallic nanoparticles is self-assembly in approximately defined nanoarchitectures [18], [19], [28]–[30].

All simulations are conducted at non-zero temperature (*T* = 5 K). At high temperatures, random electron tunneling makes it difficult to implement reliable information processing schemes for the system parameters mentioned above (1 aF for the NP capacitance and 10 GΩ for the charging resistance). However, higher temperatures can be considered by decreasing further the electrical capacitance, which can be achieved by reducing the NP size and introducing appropriate organic ligands [2], [18]–[20], [22], [29], [30]. Recently, Coulomb blockade effects have been observed up to 160 K in ligand-stabilized Au NP with nanoscale diameters [2]. Operation at room temperature [19] has been demonstrated with chemisorbed Au NP of 1.8 nm core diameter and scanning tunneling spectroscopy techniques. Silicon-based MOS-SET nanodevices suffering from a significant variability have also been operated at 300 K [3]. In any case, the threshold variability effects analyzed here are rather general and should be observed experimentally regardless of the characteristic temperature of the particular type of nanostructure considered.

### Charging equations of an oscillatory R-SET

The building block *i* of a R-SET ensemble (Fig. 1A) is composed of a NP linked to an electrode (ground potential) by a ligand acting as a tunneling junction. The ligand-protected NP behaves as a SET and its equivalent circuit consists of a capacitance *C_i_* in parallel to a tunneling junction of resistance *R_i_* [23]. A high resistance *r_i_* connects the SET to another electrode at potential *V* to form the R-SET.

At low temperatures, the Coulomb blockade gives the relaxation oscillation of Fig. 1A. The time variation of the potential *V_i_* of the NP *i* due to the charging process can be described approximately by the equation [24].
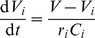
(1)


and depends on the time *r_i_C_i_* and the applied potential *V*. The potential *V_i_* of the NP *i* increases towards *V* because of the charging process. The oscillations are observed when *V* is close enough to the NP threshold potential *V_i,_*_th_ = *e*/2*C_i_*, where *e* is the elementary charge. For *V*<*V_i_*_,th_, the NP potential tends asymptotically to *V* but no oscillation occurs.

Tunneling can be described as a stochastic process of rate [24].
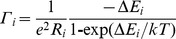
(2)when the tunneling junction resistance *R_i_* is much higher than the quantum resistance *R_h_* = *h*/2*e*, where *h* is the Planck constant, *k* is the Boltzmann constant, and
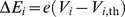
(3)is the change in electrostatic energy because of the electron tunneling. This tunneling event makes *Vi* to decrease by -2*Vi*,th and, subsequently, the charging-tunneling process is resumed. Eqs. (1) – (3) describe the charging–tunneling process of R-SET *i*. According to the above section, typical values of the R-SET parameters are *Ci* = 1 aF, *Ri* = 10 MΩ, and *ri* = 10 GΩ, so that the time constant for the charging process is of the order of *riCi* = 10 ns. The charging resistance is much higher than the tunneling resistance, 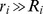, to ensure a relatively stable charging period.

To simulate the charging–tunneling process, a mixed continuum–Monte Carlo approach is used [24]. Initially the NP potential is set to zero (*V_i_* = 0) and the NP charging progresses according to Eq. (1). At a fixed time step, 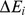 is estimated using Eq. (3) and a Metropolis algorithm is used to evaluate the probability of the electron tunneling from the ground electrode to the NP. From this probability, a decision is made. If tunneling occurs, then the NP potential is decreased by -2*V_i_*_,th_ and the charging process is resumed. The tunneling event is considered to be instantaneous with respect to the average charging time. The periodic sequence of charging-tunneling events produces the electrical potential spikes of Fig. 1A which are reminiscent of those found in the integrate-and-fire neuronal models [17], [21], [24].

### Nanostructure variability in heterogeneous ensembles

The simulations take into account the experimental fact that nanostructures show statistical distributions of physical parameters characterized by their experimental average and width distribution values. Our objective is to obtain the integrated response of an ensemble of R-SET showing a significant heterogeneity in the individual physical properties. The nanostructure variability is incorporated by considering random distributions of relative width *δ* for the NP capacitances and the charging resistances [15] (the effects of considering different distributions have recently been analyzed in Ref. [31]). The central values of these distributions are 1 aF and 10 GΩ, respectively. From previous studies, the relative variability *δ* = 0.25 is considered to be representative of the experimental threshold-potential nanostructures [2], [3], [15], [18], [19]. Thus, the capacitance of the NP assumes random values between 0.75 aF and 1.25 aF while the charging resistance of the R-SET takes random values between 7.5 GΩ and 12.5 GΩ. The collective response of a homogeneous ensemble with *N* identical R-SET (*δ* = 0) is also evaluated to show the effects of variability.

### Processing an image

The frequency of tunneling events (the spikes of Fig. 1A) is related to the charging resistance, the NP capacitance, and the applied potential which allows a frequency-dependent image processing using an ensemble of R-SET [16]. First, every pixel of an image with 256 grey levels is translated into an input potential *V* = *V*_black_ + *g*_in_ *V*_white_ (Fig. 1A), where *g*_in_ is the grey level of the pixel (*g*_in_ = 0 for black and *g*_in_ = 1 for white), *V*_black_ is the potential for a black pixel, and *V*_black_ + *V*_white_ is the potential for a white pixel. This input potential is then applied to the array of parallel R-SET of Fig. 1A where each nanostructure *i* follows periodic charging-tunneling processes for *V*>*V_i_*_,th_. The frequency of the spikes generated by this particular R-SET is proportional to the input potential *V* (and then to the grey level) common to all R-SET in the array [15], [16]. An average frequency characteristic of the ensemble as a whole can be obtained by dividing the total number of spikes generated by the R-SET ensemble of Fig. 1A by the time *τ* assigned to the processing of each pixel (Fig. 1B).

Two methods have been considered to process the grey level image differing in the processing times allowed. The first method is reminiscent of the *rank order* coding previously used in neuronal models [13] and assigns an output grey level which is proportional to the number of R-SET in the ensemble that have produced a spike. The processing time must be short enough to restrict the number of spikes per individual R-SET to be one at most. Alternatively, the second method (*rate* coding) assigns an output grey level proportional to the average frequency of spikes [16]. The processing time is now high enough to allow each R-SET to spike up to *N*_grey_ times on average for the maximum input potential (*V* = *V*_black_ + *V*_white_), where *N*_grey_ is the number of grey levels used to reproduce the image.

### Processing a time dependent input signal

We consider heterogeneous and homogeneous ensembles of R-SET processing the sinusoidal input signal
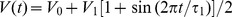
(4)where *V*_0_ is the minimum applied potential and *V*_1_/2 and 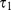 are the amplitude and the period of the signal, respectively. The ensemble output is obtained by dividing the processing time into time bins of 30 ns and counting the total number of spikes produced in the ensemble for every time bin. This procedure is followed for different input signal frequencies to better show the differences between the heterogeneous and homogeneous ensembles.

The ensemble response to *V*(*t*) is influenced by dynamic (thermal) and static (threshold diversity) noises. The thermal noise acts as a white noise whose amplitude is proportional to temperature (see Eq. (2)). This noise is incorporated in the Monte Carlo algorithm used in the simulations and, at the low temperatures used here, has a small effect compared with the threshold diversity. The input potential of Eq. (4) is noiseless because we wish to concentrate on the system diversity. The effect of adding a noise directly to the input potential was considered previously in Ref. [31].

### Synchronization of SET ensembles with heterogeneous nanostructures

Neuronal activity in the brain involves the synchronization of neuronal ensembles as a response to a common input signal. We study here the synchronization of ensembles with identical and non-identical R-SET coupled by a common resistance *r*_c_. When a coupling resistance *r*_c_ is incorporated between the electrode at potential *V* and the charging resistances of the *N* R-SETs in the ensemble, the charging process of the NP *i* is coupled to those of the other NP according to the equation [24].
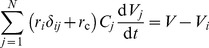
(5)where *δ_ij_* is the Kronecker delta and *V* is the input potential *V*. The coupling strength is characterized by the dimensionless parameter.



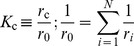
(6)The synchronization of the R-SET ensemble is characterized by the in-phase oscillations of the different NP potentials. We define the oscillation phase of NP *i* by first identifying the times 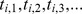 corresponding to the local maxima of the NP potential. The time between two consecutive maxima, 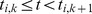, allows evaluating the phase as 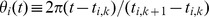. Note that the time periods 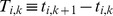 are not equal for all the oscillations of the R-SET *i* because of the coupling with the other R-SET and the thermal noise.

The complex order parameter
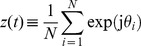
(7)where 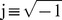 characterizes quantitatively the synchronization of the ensemble and it is determined by the coupling strength parameter of Eq. (6). The modulus 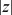 of the order parameter tends to 1 for full synchronization and to 0 for a large number (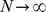) of uncorrelated oscillators [24].

## Results and Discussion

### Processing an image

Fig. 1B shows the spike rate (average frequency of the oscillations) obtained with heterogeneous and homogeneous ensembles having the same number *N* = 64 of R-SET (Fig. 1 A) as a function of the applied voltage. The curves show the average spike rates calculated from 100 simulations with the different ensembles whereas the shadow region shows the maximum and minimum values obtained in these simulations. When the processing time is as low as 30 ns, typically only one spike per R-SET occurs (note that the charging time *r_i_C_i_*≈10 ns) regardless of the nanostructure variability. The heterogeneous and homogeneous ensembles of nanostructures show a significantly different response in Fig. 1B [16]. For identical R-SET (*δ* = 0) a binary-like response with a sharp transition is observed: none of the R-SET spikes for low (sub-threshold) potentials while all of them spike for high (supra-threshold) potentials. The transition region with smoothing corners around the threshold potential (80 mV approximately) is due to the thermal fluctuations. Contrarily, a graded response is obtained for *δ* = 0.25 because of the diversity in the threshold potentials of the R-SET in the ensemble. When the processing time is increased to 120 ns, the ensemble of non-identical R-SET shows a qualitatively similar behavior, but the ensemble of identical R-SET exhibits then a stair-like response because the increase in the processing time allows each R-SET to complete several charging-tunneling cycles [16].

**Figure 2:**
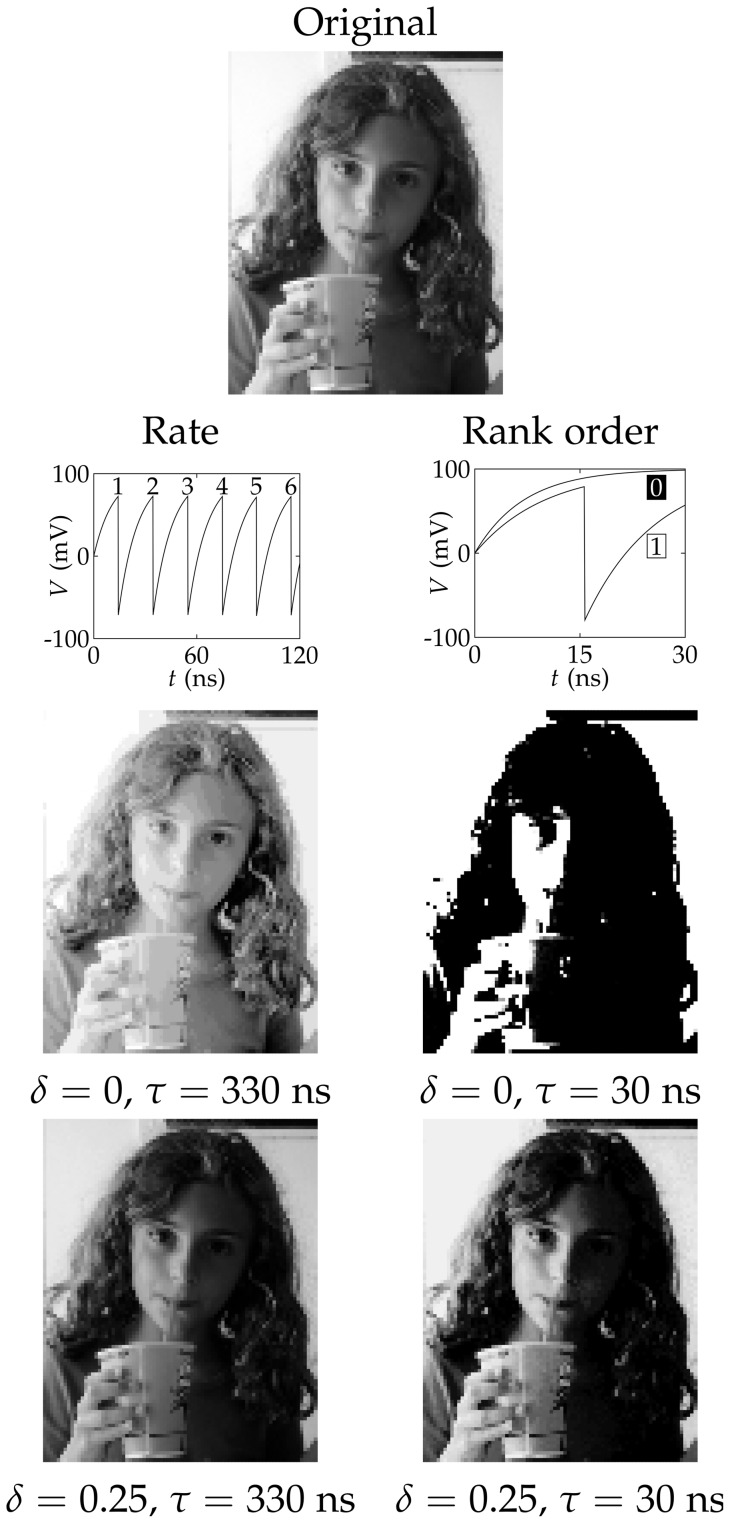
Rate and rank order image processing. Processing of a grey image by homogeneous (*δ* = 0) and heterogeneous (*δ* = 0.25) ensembles of R-SET using the rate coding (left) and the rank order coding (right) schemes. The rate coding employs the spike frequency calculated as the number of spikes divided by the time window (the spike frequency is 6 spikes in 120 ns or 50 MHz in the example of the figure). This coding assigns a grey level to the average spike frequency of the ensemble. On the contrary, the rank order coding uses a 0/1 response, where 0 indicates that no spike occurred while 1 indicates that a single spike occurred in the time window (30 ns in the example of the figure). This coding assigns a grey level to the number of R-SETs that spiked (response 1).

Fig. 2 shows the image processing with the above nanostructure ensembles using two different coding schemes. In both cases, the 256 grey levels in the pixels of the image (Fig. 1A) are linearly transformed to input potentials and applied to an ensemble of *N* = 64 R-SET. The response of the ensemble is then followed during a processing time *τ* for every pixel. The *rate* coding transforms the input potentials of the different grey levels into average spike rates. These rates are evaluated for a sufficiently long processing time allowing several spikes per R-SET in the ensemble. This scheme has been studied recently [16] and it is presented here for comparison with the *rank* order coding. In our implementation, the rank order coding evaluates how many of the R-SET in the ensemble have spiked during a relatively short processing time (compared with that of the rate order coding). The grey levels of the output image are now recovered according to the number of nanostructures in the ensemble that have spiked (a *no spike/one spike* response is obtained for every R-SET because of the relatively short processing time allowed in this case). This implementation of the rank order coding differs from that of Ref. [13] which is based on the order of firing in the cells rather than on the number of the nanostructures that have produced a spike.

The results obtained with the two coding schemes are shown in Fig. 2 for the same number *N* = 64 of non-identical and identical R-SET. The ensemble average values of the charging resistance (10 GΩ) and NP capacitance (1 aF) are the same as those of Fig. 1B. We take *V*_white_ = 100 mV and *V*_black_ = 60 mV, except for case of the rate coding by identical R-SET (*δ* = 0) where we change *V*_black_ to 75 mV in order to avoid the potential region where the ensemble shows no response (between 50 mV and 65 mV in the inset of Fig. 1B). The processing time allowed for each pixel is different in the two schemes. The rate coding takes a long time (330 ns) to allow the homogeneous ensemble to gather enough information to process the image. Note that the average spike rate for an ensemble of identical R-SET follows the stair-like dependence with the voltage of Fig. 1B. A long processing time allows for a large number of steps in the stair and this fact results in a large number of grey levels in the processed image. In Fig. 2, the selected time allows for *N*_grey_ = 16 grey levels in the processed image out of the 256 levels in the original image. The processing time allowed in the rank order coding (only 30 ns) is shorter than in the rate order coding because a *no spike/one spike* response for every R-SET in the ensemble is needed.

Fig. 2 shows that the rate coding can process the image for both *δ* = 0 and *δ* = 0.25 although a close inspection reveals a better image reconstruction in the case of the heterogeneous ensemble (*δ* = 0.25) [16]. The graded response of the heterogeneous ensemble with the input potential gives an extended dynamic range (Fig. 1B) and this result is advantageous here. However, it is in the rank order coding where the difference between homogeneous and heterogeneous ensembles becomes obvious. The rank order coding is a limiting case of the rate order coding and, because all the nanostructures in the ensemble behave similarly when they are identical (*δ* = 0), the output image contains only full white and black pixels (there is some small grading due to the thermal fluctuations at non-zero temperature). On the contrary, the different individual thresholds of the non-identical R-SET in the heterogeneous ensemble allow a better image reconstruction even in the limiting case of *no spike/one spike* response. The image processing is also improved when a larger ensemble is used or a longer time is allowed (the latter case would eventually transform the rank order coding into the rate order coding).

The oscillating phenomena observed in R-SET ensembles are reminiscent of those found in models of integrate-and-fire neurons [16], [26]. Although the ensembles of nanostructures considered here lack the complex characteristics of neuronal networks (in particular, the synaptic weights producing different levels of neural actuation) [12], [13], the results of Fig. 2 clearly suggest that the diversity of sensing units constitutes an important advantage for processing weak analog signals [7], [10]. In particular, the diversity of threshold potentials should allow for a rapid but still reliable image reconstruction in the case of the rank order coding with heterogeneous ensembles. The approximate but fast recognition of an input image may constitute a survival strategy in a rapidly changing environment [32] and it has been cited that human visual processing is too fast to be based on rate coding algorithms involving the long time collection of many individual firing rates [13]. More efficient ways of encoding an image with only a few spikes have been proposed (e.g., the order of firing in the cells of the visual system [13]). In this sense, exploiting the variability inherent to threshold potential nanostructures (Figs. 1 and 2) can be considered a biologically-inspired strategy.

### Processing a time dependent input signal

**Figure 3:**
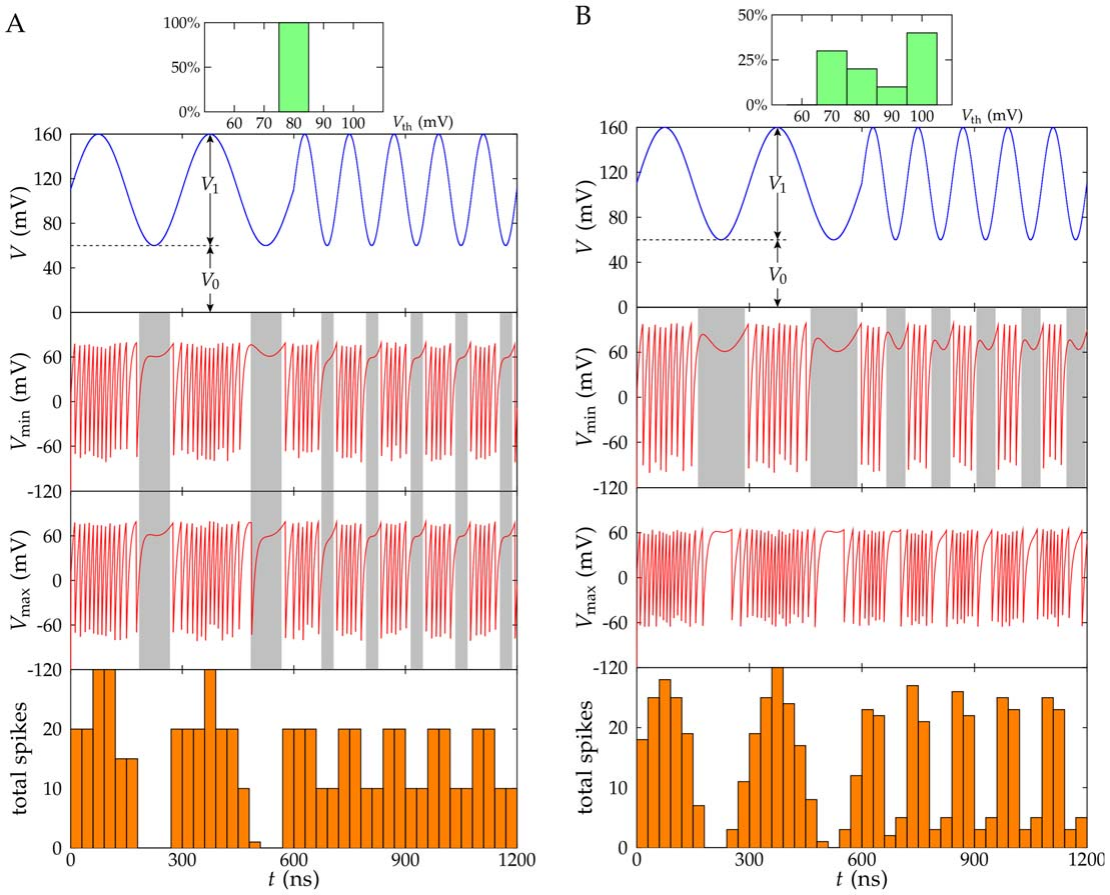
Processing analog signals in the supra-threshold regime. The responses of ensembles with *N* = 10 identical (A) and non-identical (B) R-SET to a sinusoidal input potential (top panel, *V*_1_ = 100 mV) that is higher than the average threshold potential *V*_th_≈80 mV for most of the time (*supra-threshold* regime). The histograms show the distribution of threshold potentials for the nanostructures. The middle panels correspond to the potentials *V*_min_(*t*) and *V*_max_(*t*) of the NP in the two R-SET that show the minimum and maximum number of spikes, respectively. The grey bars in the middle panels indicate the time regions where the input potential is lower than the particular threshold potential of the R-SET. The bottom panel shows the total number of spikes in the ensemble for time bins of 30 ns. Note that although all R-SETs have the same threshold potential when *δ* = 0 (A), the potentials of two individual NP in the ensemble can still be slightly different because of the thermal noise.

**Figure 4:**
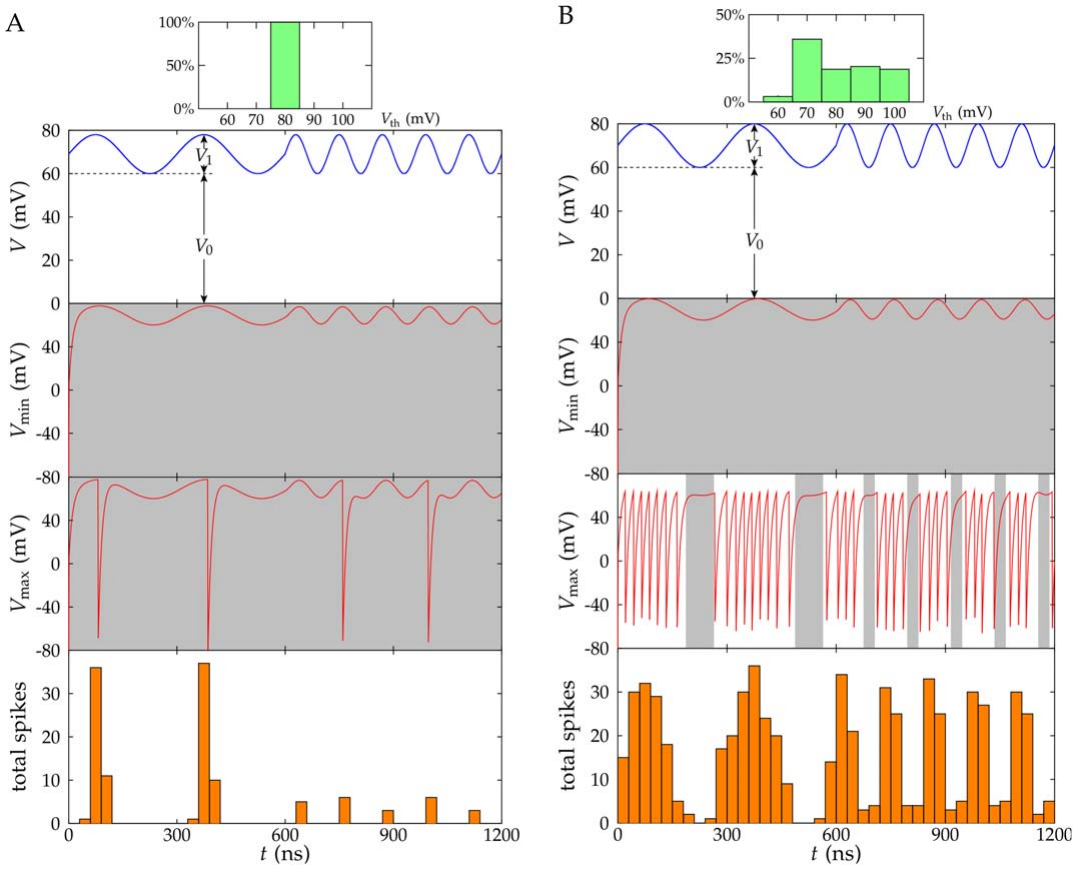
Processing analog signals in the sub-threshold regime. The same problem shown in Fig. 3 but now for *N* = 64 and *V*_1_ = 18 mV, so that the ensemble operate most of the time in the *sub-threshold* regime. Although the total number of spikes in Fig. 4B is larger than in Fig. 3B, the average number of spikes per R-SET is smaller in Fig. 4 (*N* = 64) than in Fig. 3 (*N* = 10), as it should be expected.

To better understand the effects of nanostructure variability on signal processing, Figs. 3 and 4 show the different response of heterogeneous (*δ* = 0.25) and homogeneous (*δ* = 0) ensembles to a time dependent signal. We take Eq. (4) as the input voltage and introduce *V*_0_ = 60 mV. We consider two different periods (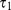 = 300 ns and 120 ns) and amplitudes (*V*_1_ = 100 mV and *V*_1_ = 18 mV) in Eq. (4) to allow for slow and fast variations in the *supra-threshold* and *sub-threshold* regimes, respectively (note that the average threshold potential is 80 mV approximately).

The effect of threshold distributions in the optimization of information transmission with ensembles of noisy elements through suprathreshold stochastic resonance has been studied previously by McDonnell, Stocks, Pearce, and Abbott. [33]. In particular, the relevance of this effect for neural population coding was emphasized [33]. Also, different models in which suprathreshold stochastic resonance occur, together with the exploitation of the resonance in particular designs concerning distributed sensor networks, nano-electronics, and biomedical applications, have been discussed with detail recently [34].

Fig. 3A and B correspond to an input potential which is over the average threshold value of the R-SET ensembles. On the contrary, Fig. 4A and B correspond to an input potential which is significantly lower than the above threshold value. The average values for the NP capacitance and the charging resistance are those of Figs. 1 and 2 while the number of R-SET in the processing ensemble has been reduced to 10 in Fig. 3 (supra-threshold regime) and increased back to 64 in Fig. 4 (sub-threshold regime).

To illustrate the different individual responses of the R-SET in the ensembles, Figs. 3 and 4 include the time variations of the NP with the minimum (*V*_min_) and the maximum (*V*_max_) number of spikes obtained during the processing time (see the two central rows). In the case of non-identical nanostructures, these curves correspond to the highest and the lowest threshold potentials in the ensemble, respectively. Finally, Figs. 3 and 4 (bottom figure) show the collective response of the R-SET ensembles obtained by dividing the processing time *τ* = 600 ns into time bins of 30 ns and counting the total number of spikes produced in the ensemble for every time bin.

The shaded regions in Figs. 3 and 4 indicate the time periods when the input potential is lower than the individual threshold potentials. Spikes are more likely observed outside these regions. As expected, in Fig. 3B the potential *V*_max_ (R-SET with the minimum threshold potential) spikes more often than the potential *V*_min_ (R-SET with the maximum threshold potential). Because identical nanostructures have the same threshold potential, the differences between *V*_max_(*t*) and *V*_min_(*t*) are small in Fig. 3A and can be ascribed to the thermal noise.

If we compare the time dependence of the total number of spikes (Fig. 3, bottom panel) with the input potential *V*(*t*) (Figs. 3, top panel), the heterogeneous ensemble (Fig. 3B) allows for a better reconstruction of the input signal than the homogeneous ensemble (Figs. 3A) in the supra-threshold regime. This result is a consequence of the threshold variability producing different individual responses that are finally combined to give the collective response of the ensemble (Fig. 3B, bottom panel). While the R-SET with minimum threshold potential spikes most of the time, the R-SET with maximum threshold potential spikes only when the input potential attains sufficiently high values. The different individual responses allow for a more reliable reproduction of the analog input signal in the case of the heterogeneous ensemble compared with the homogeneous ensemble (Figs. 3A and B, bottom panel).

When the frequency of the signal is increased (Fig. 3A, top panel) the ensemble with *δ* = 0 only shows two states which correspond to the peaks and valleys of the input signal (Fig. 3A, bottom panel). In this case, only the frequency (not the shape) of the input signal can be detected. This problem is not so apparent for the ensemble with *δ* = 0.25 (Fig. 3B, bottom panel) where the diversity of individual responses allows for more intermediate states.

Fig. 4 corresponds to the sub-threshold regime where the amplitude of the input signal is reduced to only *V*_1_ = 18 mV and the number of R-SET in the ensemble has been increased to 64 to improve the resolution (the rest of parameters have the same values as in Fig. 3). The weak input signal deteriorates the response of the ensemble with identical R-SET (Fig. 4A, bottom panel), as it is clearly shown by the potentials *V*_min_(*t*) and *V*_max_(*t*) of the NP with minimum and maximum number of spikes (Fig. 4A, middle panel). In this case, many of the R-SET do not spike because their NP potentials are significantly lower than the threshold potential. The ensemble of identical nanostructures is unreliable in terms of inferring information about the input signal (Fig. 4A, top and bottom panel).

The situation changes dramatically when we consider the heterogeneous ensemble. Although the individual responses of R-SET are still unreliable (Fig. 4B, middle panel), the collective output of a sufficiently large ensemble closely reproduces the sub-threshold input signal (Fig. 4B, bottom panel). Indeed, the heterogeneous ensemble gives a response for sub-threshold signals (Fig. 4B, bottom panel) which is similar to that obtained for supra-threshold signals (Fig. 3B, bottom panel) provided that we increase the number of R-SET from 10 to 64. The increase in the number of basic units of the ensemble (system redundancy) not only compensates for the decreased output response due to the diversity but also permits to extend the dynamic response of the heterogeneous ensemble with respect to that of the homogeneous ensemble. This result, which is based on the variability of the individual responses, provides some clues of why the nervous system uses diversity in detection cells (see in particular Fig. 2 of Ref. [10]) and suggests that intrinsic neuronal variability serves a function and is not merely a reflection of biological imprecision [7].

### Synchronization of SET heterogeneous ensembles

**Figure 5:**
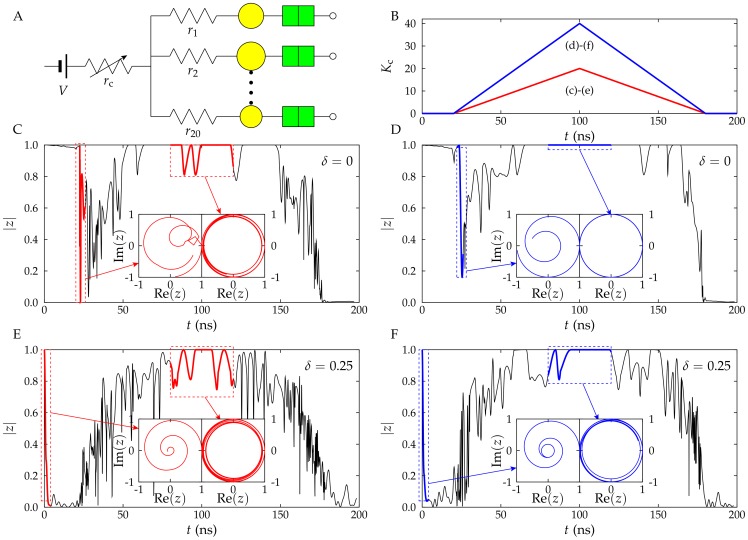
Synchronization of R-SET ensembles. Synchronization of R-SET ensembles for an applied potential *V* = 120 mV (A). The coupling resistance *r*_c_ (and then the coupling strength *K*_c_) changes with time following a triangular signal (B). The modulus of the order parameter 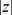 describing the synchronization shows different time variations for identical, *δ* = 0 (Fig. 5C and D), and non-identical ensembles, *δ* = 0.25 (Fig. 5E and F). The low and high synchronization states are highlighted in the insets showing the imaginary and real parts of *z*. A high degree of synchronization is achieved by increasing the maximum value of *K*_c_ from 20 (Fig. 5C and E) to 40 (Fig. 5D and F). We assume that the ensembles are initially (*t* = 0) synchronized and desynchronize because of the thermal noise and the initial zero value of the coupling strength (Fig. 5B). Synchronization is established when the coupling strength reaches sufficiently high values. Finally, the ensembles desynchronize again when the coupling strength decreases back to zero (Fig. 5B).

The dynamic formation of ensemble synchronized states can also be used as a temporal coding mechanism because firing synchrony is a collective characteristic which is robust to individual failures. Synchronization could then be used to detect particular events and process temporal data series. Therefore, it is useful to compare the synchronization and desynchronization processes in two ensembles of *N* = 20 identical and non-identical nanostructures (Fig. 5A) with a coupling resistance that changes with time as shown in Fig. 5B. The coupling parameter increases from *K*_c_ = 0 to a maximum value *K*_c_ = 20 (Figs. 5C and 5E) or 40 (Figs. 5D and 5F) and back again to *K*_c_ = 0. The synchronization and desynchronization processes of two ensembles of 20 identical (Fig. 5C and D) and heterogeneous (Fig. 5E and F) nanostructures with the average values for the NP capacitance and charging resistance of Figs. 3 and 4 are also shown. The synchronization is evaluated using the modulus of the order parameter *z* [24]. The real and imaginary parts of *z* are shown in the insets of Fig. 5C-F for some regions where the initial desynchronization process occurs and the high synchronization state is established.

The degree of synchronization is evaluated from the phases of the individual oscillators that can be calculated from the time between two consecutive maxima (Figs. 1 and 3). This period depends on the charging resistance and the NP capacitance of each individual R-SET (Eq. (1)). The NP potentials of all R-SET are set to *V_i_* = 0 initially, so that the nanoscillators are synchronized at *t* = 0. The desynchronized state is only observed when a small interaction between the R-SET of Fig. 5A is allowed (*K*_c_ increases from zero, Fig. 5B). The desynchronization process also occurs at the end of the coupling strength cycle where *K*_c_ tends to zero again (Fig. 5B). A clearly different behavior for the ensembles of identical (Figs. 5C and D) and non-identical (Figs. 5E and F) nanostructures is observed in the initial time region (20 ns) with *K*_c_ = 0 (Fig. 5B). When *δ* = 0, the thermal noise is the only factor that may drive the desynchronization process. This process is then so slow that 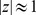 after 20 ns. On the contrary, in the heterogeneous ensemble (*δ* = 0.25) the variability in the individual parameters of the R-SET drives quickly the initial desynchronization process (Figs. 5E and F).

Synchronization occurs over a central time window which is more extended for the identical (Fig. 5C and D) than for the non-identical (Fig. 5E and F) nanostructures. Although the diversity makes more difficult the synchronization of the heterogeneous ensemble (i.e., a higher value of the coupling strength is needed for *δ* = 0.25 than for *δ* = 0 to achieve the same degree of synchronization), synchronization is also possible even for significantly different nanostructures (Figs. 5E and F). As expected, synchronization is enhanced by increasing the maximum value of the coupling strength from *K*_c_ = 20 (Fig. 5C and E) to 40 (Fig. 5D and F). On the contrary, when the coupling strength maximum is decreased to *K*_c_ = 10 (data not shown), full synchronization is not achieved.

Full synchronization (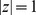) is achieved when one particular R-SET dictates the potential phase of the whole ensemble. This occurs when this nanostructure becomes the commanding R-SET and sets the ensemble synchronous period to be equal to its particular charging–tunneling time [24]. A high degree of synchronization is obtained when the coupling strength takes the maximum value although some fluctuations are still apparent because a particular R-SET may experience tunneling at a charging time different to that of the commanding R-SET [24].

It is tempting now to relate some of the above results with those found in biophysical networks. Synchronization of neural networks is usually analyzed using integrate-and-fire models that incorporate coupling schemes more complex than that considered here [35]. The dynamic formation of synchronized neuron clusters has been proposed as a temporal coding mechanism [32] that could be used to detect particular events and analyze temporal data series. There is also some evidence that both positive and negative changes in the degree of synchronization can be relevant signals for neuronal information processing [14].

However, synchronized states may constitute stable attractors difficult to reset [36] which should be undesirable for the efficient processing of rapidly changing environmental conditions [14]. Fig. 5C and D show indeed that homogeneous ensembles are rapidly synchronized when the coupling strength *K*_c_ increases but they are also difficult to desynchronize again when *K*_c_ decreases. If the *reset* function is not easily implemented, a rapid transition between the different ensemble states is not possible, and this fact makes difficult to follow the time dependent input signals. This characteristic of the homogeneous ensemble is thus a serious limitation for information processing. On the contrary, the heterogeneous ensemble permits rapid transitions between the synchronized and desynchronized states because of the effects of the nanostructure variability and the thermal noise on the collective response. High synchronization degrees could be present in some pathological states of neural networks [8]. Although clusters of coupled neurons in the brain show transient synchronization patterns that are certainly much complex than those observed here for R-SET nanostructures, the results of Fig. 5 provide some clues on the crucial role of biophysical diversity and noise in biological networks.

## Conclusions

Nanotechnology is bound to produce ensembles of structures that show a significant variability in their individual physical properties. This fact constitutes a serious problem because nominally identical units are required in many practical applications. In particular, this is the case of information processing. However, the results obtained in Figs. 1, 2, 3, 4, 5 clearly show that the reliable processing of weak signals is still possible using heterogeneous groups of non-identical threshold potential nanostructures provided that some conceptual schemes typical of biological networks are invoked.

A possible experimental realization of a SET is the nanoparticle-based transistor that mimics some of the spiking characteristics of neurons. We have simulated the integrated response of ensembles of SET that suffer from a high individual variability. The ensembles are not modeled as replicates of the same nanostructure with constant physical properties but as statistical distributions of physical parameters characterized by some average and width distribution values. We consider simple but general information processing schemes to draw conclusions that should be of relevance for other threshold-based nanostructures. Monte Carlo simulations suggest that ensembles of heterogeneous nanostructures whose physical parameters follow statistical distributions approximately controlled could be more efficient than those with identical units because of the extended dynamic response. The integrated ensemble response is based on collective phenomena robust to individual failures and variability.

The three case studies considered here permit to understand the effects of nanostructure variability in a broad range of information processing schemes that could also be relevant for biophysical systems. In particular, the neural variability and its functional significance, together with the role of stochastic resonance and noise in the nervous system, have been discussed recently [5]–[11], [37]. Heterogeneous ensembles of nanostructures can process weak, sub-threshold signals, permit rapid transitions between different synchronization states, and display some of the characteristics previously observed in neural networks. The results of Figs. 1, 2, 3, 4, 5 suggest that the role of neural diversity in the complex cognitive tasks of brain could constitute a possible source of inspiration for the design of simple information schemes with artificial threshold nanostructures showing a high variability.
